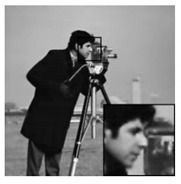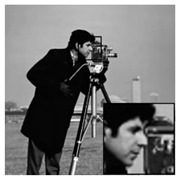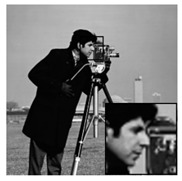# Fuzzy Adaptive-Sampling Block Compressed Sensing for Wireless Multimedia Sensor Networks

**DOI:** 10.3390/s20216217

**Published:** 2020-10-31

**Authors:** Sovannarith Heng, Phet Aimtongkham, Van Nhan Vo, Tri Gia Nguyen, Chakchai So-In

**Affiliations:** 1 Department of Computer Science, Faculty of Science, Khon Kaen University, Khon Kaen 40002, Thailand; sovannarith@rupp.edu.kh (S.H.); phet@kkumail.com (P.A.); nguyengiatri@duytan.edu.vn (T.G.N.); 2 Department of Computer Science, Faculty of Science, Royal University of Phnom Penh, Phnom Penh 12156, Cambodia; 3 International School, Duy Tan University, Danang 550000, Vietnam; vonhanvan@dtu.edu.vn; 4 Institutes of Research and Development, Duy Tan University, Danang 550000, Vietnam

**Keywords:** adaptive sampling, block compressed sensing, feature selection, fuzzy logic system, wireless multimedia sensor networks

## Abstract

The transmission of high-volume multimedia content (e.g., images) is challenging for a resource-constrained wireless multimedia sensor network (WMSN) due to energy consumption requirements. Redundant image information can be compressed using traditional compression techniques at the cost of considerable energy consumption. Fortunately, compressed sensing (CS) has been introduced as a low-complexity coding scheme for WMSNs. However, the storage and processing of CS-generated images and measurement matrices require substantial memory. Block compressed sensing (BCS) can mitigate this problem. Nevertheless, allocating a fixed sampling to all blocks is impractical since each block holds different information. Although solutions such as adaptive block compressed sensing (ABCS) exist, they lack robustness across various types of images. As a solution, we propose a holistic WMSN architecture for image transmission that performs well on diverse images by leveraging saliency and standard deviation features. A fuzzy logic system (FLS) is then used to determine the appropriate features when allocating the sampling, and each corresponding block is resized using CS. The combined FLS and BCS algorithms are implemented with smoothed projected Landweber (SPL) reconstruction to determine the convergence speed. The experiments confirm the promising performance of the proposed algorithm compared with that of conventional and state-of-the-art algorithms.

## 1. Introduction

The wireless sensor network (WSN), which is composed of a large number of tiny resource-constrained wireless sensor nodes (SNs), has become a pervasive emerging technology [1]. WSNs can be deployed for use in various fields, e.g., military applications, disaster management, industry, environmental monitoring, and agricultural farming [2]; consequently, they have received considerable attention from the research community [3] and have become a pillar of the Internet of Things (IoT) [4]. Challenges such as routing and clustering, security and privacy, and localization and coverage [1,2,3,5,6] have been investigated. However, most of the related studies have focused on the collection of scalar data (e.g., temperature, pressure, humidity, or object locations), followed by the transmission of the collected data via low-bandwidth data streams to a base station (BS) [3].

The advent of inexpensive hardware, such as complementary metal-oxide-semiconductor (CMOS) cameras and microphones, that can be integrated with SNs has allowed multiple types of sensors to be combined to construct wireless multimedia sensor networks (WMSNs) [4]. As a result, the focus of researchers studying WSNs is also shifting towards WMSNs. The goal is to enable the efficient transmission of not only scalar data but also multimedia streams, such as still images and video and audio streams [3,6]. Similar to WSNs, WMSNs are resource-constrained networks, and multimedia data are naturally large in size; therefore, many challenges exist in processing and transmitting multimedia content over resource-constrained WMSN networks because SNs typically have limited capacities in terms of processing power, memory, battery lifetime, and throughput. Furthermore, during wireless transmission, WMSNs also suffer from problems such as interference, multipath fading, shadowing and high signal attenuation, which can cause high bit-error rates and packet congestion [7].

Fortunately, images contain large amounts of redundant information that can be exploited to reduce their volume [8]. Traditional image compression techniques (e.g., JPEG, JPEG2000, and SPIHT), which are based on the Nyquist sampling theorem, can substantially reduce the size of an image while ensuring satisfactory image quality; however, these methods are unsuitable for implementation in single SNs because they are complicated and easily affected by channel errors during transmission. For instance, losing even a few bits during image transmission can threaten the success of the reconstruction process [9].

To mitigate this problem, compressed sensing (CS) was recently presented. Developed in 2004, CS has recently gained considerable attention from researchers because it can be used to compress multimedia data such as images and videos effectively [9,10,11,12,13,14,15,16,17]. Moreover, in the fields of data compression and communication, CS is one of the best theories due to its performance and nonadaptive coding, and its encoding and decoding operations are independent [18]. CS also combines image acquisition and image compression into a single process that does not require a raw image [19]. Since CS requires fewer samples than do the conventional methods, the amount of data collected can be drastically reduced. Moreover, CS is resistant to transmission errors because the image reconstruction process is only slightly affected by sampling losses during image transmission [14]. Therefore, CS is suitable for image acquisition and processing with massive data in WMSNs. However, the image and measurement matrices generated by CS still require large amounts of storage space and high computational power.

Block compressed sensing (BCS) has been presented to address these problems [20]. In BCS, the original image is divided into equal-sized nonoverlapping blocks to reduce memory consumption and complexity. Each image block is then processed individually using the same fixed measurement matrix and reconstructed independently at the decoder, typically a BS. However, allocating the same fixed sampling to all blocks without considering the feature information in each block is impractical, because not all blocks in an image contain the same useful information.

Therefore, adaptive block compressed sensing (ABCS) algorithms have been proposed [9,14,16,21,22] to address the issues of BCS. These ABCS algorithms adaptively allocate the sampling to each block based on an image feature, i.e., either saliency, standard deviation, edge, or texture. Sun et al. [9] exploited texture features to adaptively sample each block in their ABCS algorithm. The texture contrast in each block was computed by measuring the texture variations of each pixel in the block. Zhang et al. [14] proposed a standard-deviation-based ABCS algorithm in which standard deviation values are utilized to allocate the sampling for each block. The authors of [16] used saliency values to adaptively determine the sampling of each block. Distinct regions in an image that attract viewers’ attention are called salient objects. The saliency value is generated from the contrast between CS measurements. Based on observation, Zhou et al. [23] introduced an irregular-block-based ABCS algorithm in which the size of each block varies based on the saliency values in each block, which are also used to determine the sampling for the CS process. Wang et al. [21] exploited differences in the texture information in images to allocate the sampling for each block. The limitation of these studies is that they used only one feature for different types of images; however, some features may impact the quality of the reconstructed image.

Note that most of the abovementioned studies considered a fixed-base sampling allocated to all blocks before additional sampling is allocated based on relevant features. However, methods based on fixed base sampling do not produce optimal results because unimportant blocks (e.g., black background blocks) are assigned the same base sampling as more important blocks.

Although various image features have been explored by these ABCS algorithms, the results show that they perform well only for specific types of images. For example, using standard deviation as a feature to allocate the sampling for each block may produce good results only for low-intensity images. Therefore, determining the adaptive sampling for each block remains a challenging problem that must be approached carefully because it can affect the quality of the reconstructed images. Although ABCS algorithms offer improved image compression efficiency, they have not considered blocking artifacts, which may lead to unsatisfactory image reconstruction quality [24]. Mun and Fowler [25] proposed a BCS-based smoothed projected Landweber reconstruction algorithm (BCS-SPL) to eliminate blocking artifacts; however, this technique can still produce unsatisfactory reconstructed images because it uses Wiener filtering and thresholding methods as well as a fixed convergence speed (limiting the number of possible iterations, which potentially affects the achievable accuracy). Note that in the fixed scheme, a high convergence speed causes greater complexity during image reconstruction, while a low convergence speed results in reconstructed images with poor quality [26].

Thus, the main challenges in developing an ABCS algorithm are to select appropriate features, assign a suitable sampling to each block based on the selected features, and adaptively determine the convergence speed of the reconstruction algorithm to yield reconstructed images with acceptable quality.

In WSNs, fuzzy logic systems (FLSs) have been utilized to enhance decision-making, reduce resource consumption, boost performance, and prolong the overall network lifetime [6]. Image data contain considerable ambiguity, and an FLS can effectively represent such uncertainty in image data. An FLS provides an excellent mathematical framework for addressing information uncertainty, and FLSs have been broadly used to model complex and high-dimensional nonlinear real-life systems [27]. FLSs can also be applied in various real-time applications and hardware implementations. For instance, the authors of [28,29] applied FLSs in a near-space hypersonic vehicle as well as in a robotic airship. An FLS is also a well-known technique for selecting appropriate weights over multiple inputs, various memberships, and outputs [6], and it is suitable for implementation in SNs [30].

Therefore, to overcome the abovementioned issue faced in ABCS, we propose a novel CS architecture that applies an FLS-based approach to the task of image transmission in WMSNs and improves the quality of the reconstructed images. Our architecture improves upon the current research by considering two types of features instead of one, a flexible base sampling and an adaptive convergence speed. The contributions of this paper are summarized below.

- We design an FLS to select suitable features that are used to adaptively assign the sampling for each block.
- We also introduce an FLS to adaptively determine the base sampling for each block.
- We enhance the reconstruction process by adaptively determining the convergence speed using an FLS.

The remainder of this paper is organized as follows. Section 2 describes the related works. In Section 3, we present the proposed fuzzy ABCS algorithm and the reconstruction architecture for WMSNs in detail. Section 4 reports the results of experiments, and Section 5 concludes this paper and suggests directions for future work.

## 2. Related Works

BCS was pioneered by Gan [20], who proposed that an original image can be divided into many square blocks of equal size and that the same sampling can then be applied to all the blocks. This BCS approach reduces both the computational complexity and the memory requirements for image processing. Nevertheless, as mentioned above, assigning the same fixed sampling to all the blocks of an image without considering the specific features in each block is not an optimal solution, because some image blocks contain crucial information, while others may not.

Recently, many ABCS algorithms have been proposed [9,13,14,16,21,22,23] to address the various issues that arise with BCS. These ABCS algorithms assign a different sampling to each block based on its features (e.g., textures, edges, standard deviation, or saliency). In [21], an ABCS algorithm that utilized gray entropy based on image texture information was proposed. Each block contains different texture information, and the image entropy is used to calculate a corresponding texture information value. This method provides good compression and improves the image quality to some degree, but the complexity at the decoder is high. Moreover, a smooth image contains less texture information; consequently, at a low sampling rate, there may be insufficient information to ensure good image reconstruction. An ABCS algorithm based on edge features was introduced in [24] that reduces image distortions, but only for selected images. Furthermore, analogous to texture, edge information can produce better results at high sampling rates (number of samples per unit time) and it can be measured in Hertz [26]) since more detailed information of an image remains.

Zhang et al. [14] proposed a standard-deviation-based ABCS algorithm (STD-ABCS) that consists of both fixed base sampling and additional adaptive sampling and involves the following steps. First, fixed base sampling is computed. Then, the standard deviation of each block is calculated to explore the feature information and to define an additional adaptive sampling strategy for each block. At the decoder, the image is reconstructed using BCS-SPL. Only the fixed base sampling is used to reconstruct the image when fast reconstruction and basic image quality are needed, while both fixed base and the additional adaptive sampling are used to achieve higher image quality. This method offers improved image reconstruction quality over the conventional BCS algorithm. However, using the standard deviation is not optimal for allocating sampling to all images because the feature information distribution in images is typically nonuniform. Moreover, the results of this algorithm are equal to or worse than those of BCS at low sampling rates. Additionally, it has been reported that STD-ABCS performs better on low-intensity images than on other types of images.

Recently, some studies [16,23,31] have focused on exploiting saliency values in images for ABCS; such algorithms are called SABCS algorithms. The salient objects in images are distinct regions that attract viewers’ attention [32]. Yu et al. [31] presented a method for extracting saliency values using the pulsed cosine transform (PCT). High sampling is applied to high-saliency blocks, while low sampling is applied to low-saliency blocks. This method enhances the image reconstruction quality compared with conventional BCS. Nonetheless, when the block size is very large, this method cannot optimally allocate the sampling to each block because a single block may contain both high and low saliency.

To address the above problem, Zhou et al. [23] proposed a saliency-based adaptive partitioning algorithm for CS. In this approach, the size of the image blocks is not fixed; k-means clustering is used to adaptively partition the image blocks based on the saliency of each block’s neighbors. The goal of this approach is to adjust the size of each block to minimize the saliency differences among blocks. Then, adaptive sampling of each block is performed based on the blocks’ saliency values. The blocks are restored to normal size before the image reconstruction process begins. This method slightly improves the quality of the reconstructed images compared with conventional BCS. Unfortunately, using k-means clustering to adaptively determine the block size becomes more complicated as the number of blocks increases. Furthermore, this method improved the performance only on selected simple images and with a very large block size.

Li et al. [16] presented a saliency-based adaptive CS method using measurement contrast. In this approach, the saliency values of an image are extracted using the contrast between CS measurements to avoid the original image sampling approach, which causes CS to lose its superiority. The authors also proposed a reconstruction algorithm called the weighted global recovery model. However, the performance evaluation showed that this method obtained better results than the compared algorithms only when the sampling rate was less than 0.5. Moreover, the abovementioned ABCS algorithms generated fixed values for the base sampling of each block before assigning additional adaptive sampling based on the relevant features. Using a fixed base sampling for each block, these methods cannot produce improved results for all image types and sampling rates.

An ABCS algorithm that uses the spatial entropy of an image was proposed in [13]. Since spatial entropy captures considerable information (such as edge and texture information) it was used in this scheme to assign the sampling for each block. To reduce the encoder complexity, a linear model was applied to reconstruct each block based on a matrix-vector product. However, the results of this algorithm showed that it performs well only on selected images.

Liu et al. [33] adopted principal component analysis (PCA) to decompose each image patch for their CS-based image-coding technique, which reduced the complexity at the encoder to that of JPEG2000. However, JPEG2000 complexity is still too high for implementation in a single SN, and because this method depended on multiple iterations, the overall computational complexity at the decoder remained high.

The authors of [10] proposed an ABCS algorithm that used the error between blocks. In this algorithm, the image is divided into equal-sized smaller blocks, and the error between each block and its adjacent blocks is then determined. A structural complexity value for each block is then assigned based on the determined errors and used to assign the sampling for each block. This algorithm improves the quality of the reconstructed images; however, finding the error between each pair of blocks requires more computational power and memory than is typically available in a single SN. Moreover, the results of this study were compared only with those of the conventional BCS algorithm.

On the decoder side, many image reconstruction algorithms have been presented to reduce the computational cost and improve the reconstructed image quality [20,34,35,36,37,38]. In [20], Gan et al. proposed a fast calculation approach for BCS in which the reconstruction algorithm involves two main processes: Hard thresholding and projection into convex sets. This approach reduces the computational complexity and improves the reconstructed image quality compared with signal reconstruction algorithms such as orthogonal matching pursuit (OMP) [39], stagewise OMP (StOMP) [40], and gradient projection (GP) [37]. However, the blocking artifacts produced by BCS remain.

Mun et al., proposed the BCS-SPL reconstruction algorithm [38], which is based on the Landweber iteration method. This algorithm uses a Wiener filter to remove blocking artifacts. Later, a multiscale variant algorithm (MS-BCS-SPL) [25] was presented as an enhanced version of BCS-SPL based on the sampling of different decompositional levels in the transform domain. However, MS-BCS-SPL is not a full CS reconstruction algorithm because the low-frequency part of the wavelet decomposition results is not measured by the random measurement matrix. However, MS-BCS-SPL can be used as a reference for research on CS reconstruction algorithms [24].

Aside from ABCS algorithms and associated reconstruction processes, FLSs are a pioneering form of computational intelligence (CI) and have been applied in many fields to improve the handling of systems in which uncertainty exists and for which only incomplete information is available [41]. Many studies have adopted FLSs in WSNs as well as in image processing due to their effectiveness at improving decision-making. Heng et al. proposed a distributed-image compression architecture for WSNs that uses an FLS; this architecture distributes image compression tasks among the members in a cluster and uses fuzzy logic to form the clusters, select the nodes to perform each distributed task, and determine the relay nodes to forward the compressed data to the BS [6].

An energy-efficient approach was proposed in [42] that utilizes an FLS to optimize the energy consumption at high bandwidths to directly benefit WMSNs. The FLS is used to determine which underutilized Wi-Fi access points should be switched off, allowing the proposed method to save energy while maintaining acceptable network performance. Hassan et al. [43] presented a hybrid method based on median filters and fuzzy logic to identify and remove the salt-and-pepper impulse noise (SPN) that appears during image acquisition or transmission.

In the abovementioned works, all the ABCS studies adopt only one feature to allocate the samplings, which causes these algorithms to lack robustness when confronted with various types of images. Some studies have also utilized fixed-base samplings, that prevent their ABCS techniques from performing better on highly rich features of images. Therefore, we propose an ABCS algorithm that adopts two well-known image features: Standard deviation and saliency for generating sampling adaptively. Moreover, we apply adaptive convergence speed for image reconstruction to improve the reconstructed image quality.

## 3. Proposed Architecture

**Figure 1:**
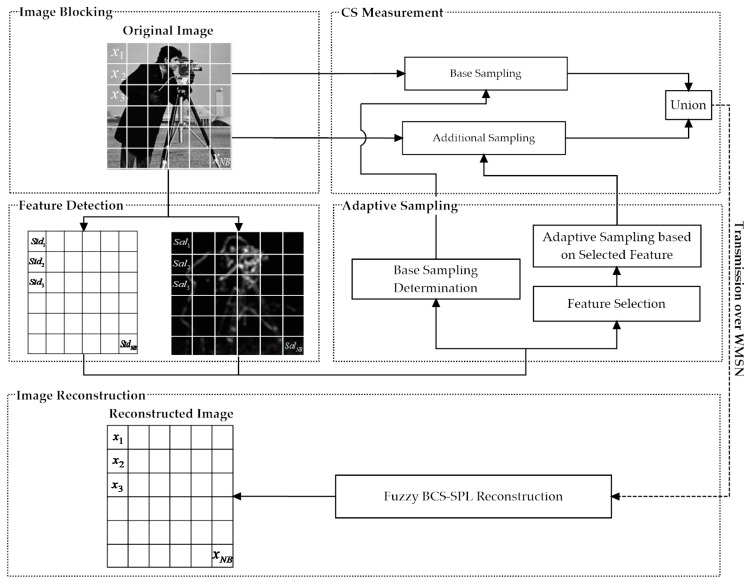
Overview of the proposed adaptive block compressed sensing (ABCS) architecture for wireless multimedia sensor networks (WMSNs).

In this section, we discuss our proposed architecture in detail, which is divided into six main phases, namely, setup, image blocking, feature detection, adaptive sampling, CS measurement, and image reconstruction, as illustrated in Figure 1.

- **Setup phase:** Our proposed scheme starts with a setup phase that generates the measurement matrix and defines some parameters. The measurement matrix and parameters are loaded into each SN before deployment to avoid wasting transmission bandwidth during the image transmission phase. This approach reduces the energy consumption and prolongs SN lifetime.
- **Image blocking phase:** After deployment, when an original image is captured by an SN, it is divided into equal-sized smaller blocks to reduce the computational complexity and memory consumption of image processing, allowing a single SN to process these blocks effectively.
- **Feature detection phase:** As soon as image blocking is complete, two types of image features, i.e., saliency and standard deviation, are computed for each block. Only one of these two feature types will be selected to allocate sampling for each block in the next phase.
- **Adaptive sampling phase:** This phase is responsible for allocating suitable sampling to each block to improve the image quality. Before the sampling is generated, an FLS is used to determine which feature will be used for calculation. This phase consists of four main subphases: Feature selection, base sampling determination, adaptive sampling determination, and oversampling adjustment, each of which is discussed in detail below.
- **CS measurement phase:** CS measurements are performed to compress the size of each block based on the adaptive sampling calculated during the adaptive sampling phase (i.e., by multiplying a random measurement matrix with a vector of the block). Then, the compressed image blocks are transmitted individually among the SNs in hop-by-hop fashion until they arrive at the BS.
- **Image reconstruction phase:** After the compressed image blocks have arrived at the BS, each block is reconstructed independently; then, the BS restores the reconstructed image from all the reconstructed image blocks. An FLS is also utilizd in this phase to calculate appropriate weights for the reconstruction algorithm to enhance the quality of the reconstructed image.

The details of all six phases are discussed in the following subsections. The notations used in this paper are shown in Table 1.

### 3.1. Setup

During the setup phase (the first phase of our proposed method) the measurement matrix that will be used for the CS process is generated, and several parameters (e.g., block size, sampling rate, and thresholds) are determined. To save bandwidth and energy for the SNs, the resulting matrix and parameters are loaded into each SN before the SNs are deployed. As a result, the SNs need to transfer only compressed images to the BS during the image transmission phase, allowing the SNs to save energy. The measurement matrix and other parameters are used both by the SNs to encode the images and by the BS to decode the images. After the measurement matrix and all the parameters have been stored in the SNs, the SNs are deployed.

### 3.2. Image Blocking

The second phase of our proposed architecture involves image blocking. After deployment, the camera-equipped SNs begin to capture images. Immediately after being captured by an SN, the original large image is divided into equal smaller blocks (e.g., 16 × 16 or 32 × 32), and each block is processed independently, as shown in Figure 1. Although image blocking degrades the image reconstruction quality, it also reduces the computational complexity and memory consumption required for image processing, allowing the resource-constrained SNs to implement CS and transmit the compressed images to their destination. Two main features must be computed for each block: Saliency and standard deviation, as discussed in Section 3.3.1 and Section 3.3.2.

### 3.3. Feature Detection

Image features are numerical values extracted from images by feature detection algorithms, and they represent the most important parts of an image. Therefore, image features play a vital role in our proposed architecture—they are used to assign a different sampling to each block. In this way, the blocks containing more important information will be allocated a higher sampling than will the less important blocks. Appropriately allocating the sampling for each block increases the quality of the reconstructed image. Based on our experiments, no single feature can produce good ABCS results for all types of images; therefore, we adopt two main types of image features (i.e., saliency and standard deviation) to allow our proposed architecture to achieve good performance on diverse images. Both types of features must be computed for each block in this phase, as depicted in Figure 1. These two feature types are explained in depth in the following subsections.

#### 3.3.1. Saliency Detection

**Figure 2:**
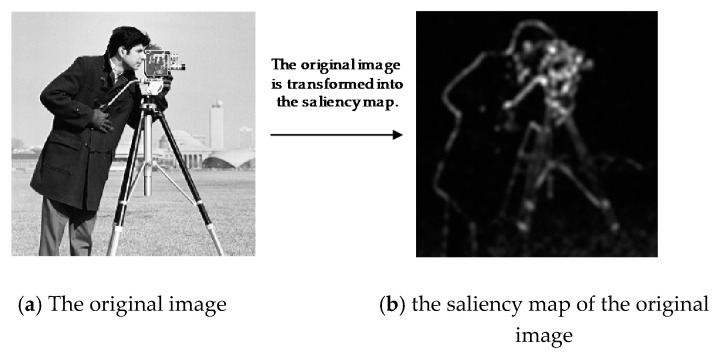
Saliency map derivation: example.

The distinct regions in an image that typically attract the attention of human viewers are called salient objects. Saliency is defined as the ability of certain parts of an image to attract human visual attention. The concept of saliency has been used for numerous research purposes in computer vision and pattern recognition [32]. As shown in Figure 2 [44], a saliency map is a two-dimensional map generated by a saliency detection algorithm to describe deviations in object shape, orientation, color, or movement relative to the environment [45]. Numerous studies have been conducted on saliency detection [31,46]. For example, Yu et al. [31] proposed a saliency detection method called the PCT, which is a simple and fast algorithm with low computational complexity suitable for saliency-based adaptive sampling in WMSNs. The PCT method captures the most highly correlated components in the visual space and uses them to indicate the salient locations. Suppose that I represents a complete image. The saliency map for I can then be computed as follows [31]:

(1) P=sign(C(I)),

(2) $$F=abs\left(C^{-1}\left(P\right)\right),$$

(3) $$SM=G\times F^{2}$$

where C and $C^{-1}$ are the 2D discrete cosine transform (DCT) and its inverse transform, respectively. The signum function is denoted by sign(.), while the absolute value function is denoted by abs(.). G is a Gaussian low-pass filter. The saliency map in Figure 2b was generated from the flower image.

Visual attention affects image perceptual quality because humans pay more attention to the salient regions. SABCS algorithms use saliency to allocate a suitable sampling for each block to improve the quality of the reconstructed image. We adopt the PCT technique for saliency detection in our proposed architecture because of its simple and rapid execution as well as its high accuracy in detecting the important objects in an image.

#### 3.3.2. Standard Deviation

The other type of feature used in our proposed method is the standard deviation. The standard deviation is a ubiquitous mathematical formula in statistics that is commonly used to represent variability or diversity. In image processing, the standard deviation represents the amount of variation or “dispersion” from an average (mean or expected value). A low standard deviation means that the data points tend to be clustered very close to the mean, whereas a high standard deviation indicates that the data points are spread over a wide range of values. The standard deviation of the i-th image block, $x_{i}$, is calculated as follows [47]:

(4) $$STD\left(x_{i}\;\right)=\sqrt{\frac{\sum_{j=1}^{N_{B}}\left(x_{i}\left(j\right)-mean\left(x_{i}\right)\right)}{N_{B}}}$$

where $x_{i}\left(j\right)$ is the value of the j-th pixel, $N_{B}$ is the size of the vector representing the block and is typically the same for all blocks, and mean(.) represents the function used to calculate the mean value. The proposed method uses standard deviation as a feature for adaptive sampling because, based on our experiments, the standard deviation feature tends to produce good results even on low-intensity images.

After both types of features have been computed for each block, the adaptive sampling phase is performed to determine which feature type should be used for the current image and how many samples should be allocated to each block based on the selected feature type. The adaptive sampling phase is described in the next subsection.

### 3.4. Adaptive Sampling

In this section, the adaptive allocation of sampling to each block is discussed in detail. This phase is divided into four subphases: Feature selection, base sampling determination, adaptive sampling determination, and oversampling adjustment. Because an FLS is adopted in this phase, we start by introducing the FLS and then discuss each subphase.

#### 3.4.1. Fuzzy Logic System (FLS)

**Figure 3:**
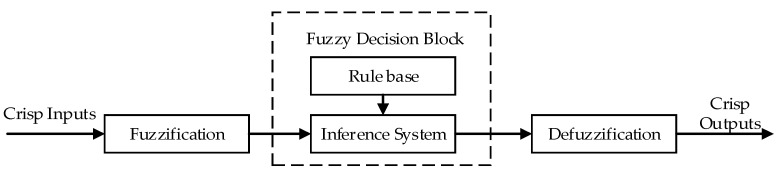
Basic block diagram of a fuzzy logic system (FLS).

Before continuing to discuss our proposed method, we briefly explain the FLS used in our architecture. Developed by L. Zadeh, fuzzy logic, or fuzzy sets, is a useful technique suitable for use in improving decision-making in resource-constrained networks (i.e., WSNs) due to its low resource consumption and effective performance [30,48]. An FLS can provide excellent solutions for many control problems by imitating the human thought process. As shown in Figure 3, an FLS has four main components: A fuzzifier, an inference engine, fuzzy rules, and a defuzzifier [49].

In the fuzzification process, the fuzzifier converts crisp inputs into a fuzzy set (described in linguistic terms such as “near,” “medium,” or “far”) using a membership function. The membership function maps nonfuzzy input values to fuzzy linguistic terms (and vice versa) to enable the quantification of the linguistic terms. To date, a number of membership functions have been developed and investigated, including triangular, trapezoidal, Gaussian, piecewise linear, and singleton functions. Researchers select appropriate membership functions based on their experience and assessments. The membership function is applied during both fuzzification and defuzzification. After the fuzzification process, an inference is made by an inference system based on a set of rules stored in a rule base. Typically, the rules are IF-THEN rules with conditions and a conclusion. Finally, the output of the FLS is defuzzified using the membership function. Defuzzification transforms the fuzzy output back into a crisp output value [50].

#### 3.4.2. Feature Selection

Based on the FLS concept explanation in the previous subsection, we continue to explain our proposed method in this section. After calculating the features of each block, the feature selection subphase is executed to discover the most suitable feature type to characterize the image. Image features provide information that can be used to determine which blocks of an image contain important information. Even so, based on our experience, good results cannot be obtained for all types of images using only a single feature type. More specifically, as mentioned above, we have found that using saliency values seems to produce better results for high-intensity images, while using standard deviation values works well for low-intensity images. Therefore, our proposed architecture selects either saliency or standard deviation features to determine the adaptive sampling for all the blocks in an image.

Before finding the most appropriate feature type for the complete image, the fuzzy cost for each block is first computed using an FLS to determine the most suitable feature type for that block. If the fuzzy cost for a block is less than or equal to a set feature selection threshold (FST), the standard deviation feature will be selected as the preferred feature for this block; otherwise, saliency will be selected. FST should be carefully defined based on experiments on various images to find the best value for all types of images. The feature selection computation for the i-th block is performed as follows:

(5) $$FSB_{i}=\{\begin{array}{c} \;\;\;\;\;\;\;\;\;STD,\;\;\;\;\;\;\;\;\;\\ Sal, \end{array}\begin{array}{c} if\;FSC\;\leq FST\\ else \end{array}$$

where STD  is the standard deviation, Sal is saliency, FSC is the fuzzy output of the FLS for feature selection, as described below in this subsection, and FST is the constant feature selection threshold.

**Figure 4:**
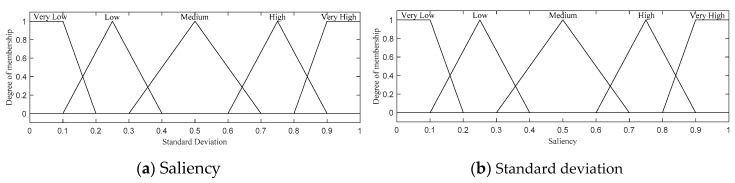
Two fuzzy input variables for feature selection.

An FLS with two input variables (i.e., saliency and standard deviation) is used to compute FSC, as depicted in Figure 4. Both input variables have the same 5 linguistic values: Very Low, Low, Medium, High, and Very High. Triangular and trapezoidal membership functions are applied to all the linguistic input values because both our experimental results and the studies in [51] show that these functions are simple and fast member functions suitable for use in WMSNs. Note that we extensively tested the performance of all possible membership functions, including triangular, trapezoidal, and Gaussian functions, and selected the best one. A similar selection process was also used for membership function selection throughout the rest of the paper.

**Figure 5:**
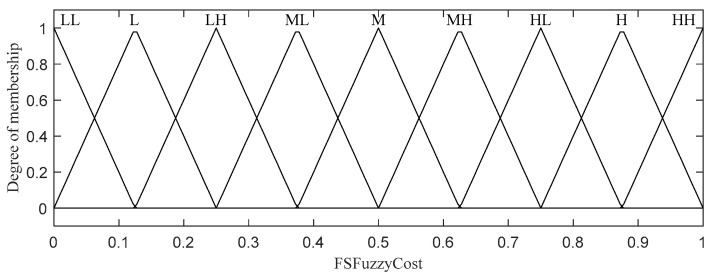
The fuzzy cost for feature selection; (LL = Low Low, L = Low, LH = Low High, ML = Medium Low, M = Medium, MH = Medium High, HL = High Low, H = High, HH = High).

A set of 25 fuzzy rules and the corresponding output membership functions are shown in Table 2 and Figure 5, respectively. Following a selection process similar to that discussed above, all the possible rules were exercised for the two input and one output variables, and the best rules were chosen.

The above computation finds the feature type most suitable for each block but not for the entire image. To find the feature type most appropriate for the entire image (all blocks), we need to compute the feature fraction, which is formulated as follows:

(6) $$FF=\;\frac{NFSB_{Sal}}{TB},$$

where $NFSB_{Sal}$ denotes the number of blocks for which saliency was selected as the preferred feature type and TB denotes the total number of blocks in the image.

Note that when FF is above a specified threshold (e.g., 0.5) meaning that the number of blocks for which saliency was selected is greater than the number of blocks for which standard deviation was selected, then saliency is chosen as the preferred feature type for the adaptive sampling of the entire image (all blocks); otherwise, standard deviation is selected. Subsequently, sampling will be allocated to all blocks based on the selected feature type. This selection process ensures that our algorithm selects the feature type that is more suitable for most blocks in the image, thereby improving the image reconstruction result. Immediately after the selected feature type is determined, the next phase is initiated to calculate the base sampling for each block.

#### 3.4.3. Base Sampling Determination

In this section, we discuss how base sampling is assigned to each block in detail. Base sampling is important because without it, some blocks with very low feature values would be allocated insufficient sampling, leading to blocking artifacts and poor reconstructed image quality. Moreover, if fast reconstruction is needed, base sampling alone can be used, whereas when better image quality is required, both the base sampling and additional adaptive sampling can be combined to improve the reconstructed image.

**Figure 6:**
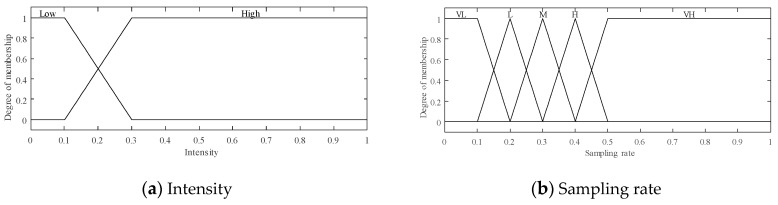
The two fuzzy input variables; (VL = Very Low, L = Low, M = Medium, H = High, VH = Very High).

In the previously mentioned ABCS studies, a fixed base sampling was assigned for all blocks without considering any features; however, a fixed base sampling alone cannot produce the best results for all types of images. Our experiments revealed that using adaptive base sampling can improve the reconstructed image quality and that the relationship between the block intensity and the sampling rate can be used to allocate the base sampling for this purpose. To allocate the base sampling for each block based on the block intensity and sampling rate, we use an FLS is used to calculate a weight that is used in Equation (8) to determine the base sampling. This FLS has 2 fuzzy inputs, namely, the block intensity and the sampling rate, as shown in Figure 6. The intensity of the i-th block is computed as follows:

(7) $$INT_{i}=\;\frac{mean\left(x_{i}\right)}{MG},$$

where $x_{i}$ is the i-th block and MG is the maximum grayscale value in the image (e.g., 255) [52]. The intensity input variable has 2 possible linguistic values, low and high, and a triangular membership function is used for both, as shown in Figure 6a. The second input variable is the sampling rate, which has 5 possible linguistic values, namely, Very Low, Low, Medium, High and Very High, as depicted in Figure 6b. A triangular membership function is used for Very Low and Very High, whereas a trapezoidal membership function is applied for Low, Medium, and High.

Note that we use Mamdani’s method as the fuzzy inference technique. We also performed an experiment using the Sugeno method but obtained lower performance; for example, in the case of the cameraman image with 0.2 sampling rates as well as 16×16 block size, the PSNR values for the Mamdani and Sugeno methods are 27.70 and 33.81, respectively.

**Figure 7:**
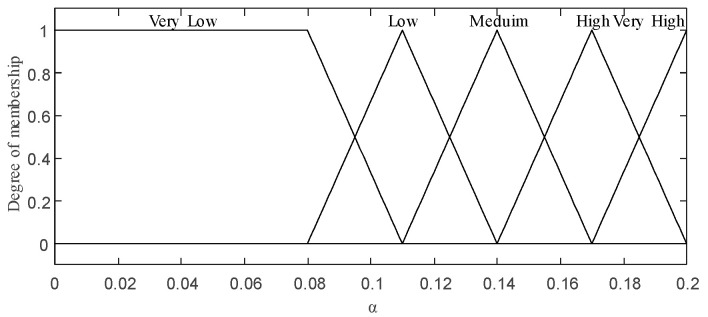
The weight; (VL = Very Low, L = Low, M = Medium, H = High, VH = Very High).

The fuzzy inference process relies on 10 fuzzy rules; these are the best rules selected from implementing all possible rules for two input variables and one output variable, as listed in Table 3. The output, depicted in Figure 7, is a weight that determines the base sampling size assigned to each block.

After the weight has been generated, the base sampling for the i-th block is calculated as follows:

(8) $$M_{i}^{0}=\;\alpha_{i}\times N_{B},$$

where α is the weight generated by the FLS as described in this section and $N_{B}$ is the block size of the image.

Next, we discuss how to allocate additional adaptive sampling for each block.

#### 3.4.4. Adaptive Sampling Determination

After the feature selection process is complete and the preferred feature type is known, additional sampling allocation is performed based on that feature type, and the results are combined with the base sampling to form the adaptive sampling for each block. The adaptive sampling for the i-th block is formulated as follows:

(9) $$AS_{i}=rnd\left(\frac{F_{i}}{\sum_{k=1}^{TB}F_{k}}\left(N-\overset{TB}{\underset{k=1}{\sum }}M_{k}^{0}\right)+M_{i}^{0}\right),$$

where $F_{i}$ is a single scalar value of the feature (e.g., standard deviation or saliency) for the i-th block, $M_{i}^{0}$ is the base sampling for the i-th block, TB is the number of blocks in the image, N is the size of the image vector, and rnd(.) is a function that rounds its argument to the nearest integer.

Immediately after adaptive sampling allocation is complete, the oversampling algorithm is executed to ensure proper adaptive sampling because—for some blocks—the number of samples allocated may be greater than the block size.

#### 3.4.5. Oversampling Adjustment

Oversampling refers to the situation in which the total sampling assigned to a block exceeds the size of the block itself. For example, the vector of a 16×16 pixel block has a length of 256 so that the maximum sampling assigned to this block should not be over 256. Even though this block is an important block, we cannot allocate more sampling to each block more than its maximum because the oversampling causes errors in the reconstruction process when the size (resolution) of the reconstructed image block is larger than that of the original block. To prevent this situation, an oversampling adjustment process is required to ensure that no oversampled blocks exist. The details of the oversampling adjustment algorithm are presented in Algorithm 1. After adaptive sampling allocation for each block, the oversampling adjustment algorithm checks the sampling for each block to determine whether it is over the size limitation of the block; if so, the oversampling is eliminated for that particular block. The total amount of oversampling from all oversampled blocks will be reassigned to nonoversampled blocks based on the feature value of each block. This process is repeated until no oversampled blocks remain. Then, the CS measurement phase, which will be discussed in the next subsection, is executed to compress each block based on the calculated adaptive sampling allocation.
**Algorithm 1** Oversampling adjustment algorithm.**Input:** Adaptive sampling: AS, Array of features of each block: F, Block size: $N_{B}$, Number of blocks: TB
**Output:** Nonoversampled adaptive sampling: $AS^{NOA}$
1: **for each**
*AS*
2: **if** the sampling in *AS* is larger than $N_{B}$
3: *OS ← 0*    //This variable is used to store the amount of oversampling among all blocks 4: *NOSI* [] *← empty* //This variable stores the indices of nonoversampled blocks 5: **for**
*counter←1* to *TB*
6:  **if**$AS\left(counter\right)>N_{B}$ 7:    $OSVal\leftarrow AS\left(counter\right)-N_{B}$ 8:    OS ← OS + OSVal 9:    AS(counter)←AS(counter)−OSVal //Subtract the excess sampling from oversampled blocks 10:  **Else** 11:   *NOSI [] ← counter*    //Add counter to the array *NOSI* 12:  **end if** 13: **end for** 14: *NOSF ← 0       //*This variable stores the total feature values of nonoversampled blocks 15: **for** counter*←*1 to count(*NOSI*) 16:   NOSF←NOSF+F(NOSI(counter))
17   **end for** 18:   **for** counter *←*1 to count(*NOSI*) 19:     $AS\left(NOSI\left(counter\right)\right)\leftarrow AS\left(NOSI\left(counter\right)\right)+\left(\left(\frac{F\left(NOSI\left(counter\right)\right)}{NOSF}\right)\times NOSF\right)$
20:   **end for** 21: **end if** 22: **end for** 23:$\;AS^{NOA}\leftarrow \;AS$


### 3.5. Compressed Sensing (CS) Measurement

Since we adopt the CS for our method, this section provides a detailed description of CS and BCS. According to conventional CS theory, an image can be recovered from a few linear measurements if there is sufficient sparsity in a specific domain. Suppose that we have a 2D image X of size $N_{1}\times$ $N_{2}$. We convert it into a column vector x of size N× *1* ($N\;=N_{1}N_{2}$) by concatenating the individual columns of X in order. The K-sparse vector contains values of one, zero, or the significant element in a specific domain (K≪N). Here, $y\in R^{M}$ is the measurement or sampling vector of x, which is computed as follows [53]:

(10) y=φx,

where φ is an M×N random measurement matrix. Based on CS theory, y can be perfectly reconstructed when M=O(KlogN).

As mentioned above, although CS offers good image compression, its implementation in a WSN faces two main challenges, namely, it requires a large amount of memory and has high computational complexity [20]. BCS was introduced to overcome these problems. In a CS architecture, the original image is divided into smaller nonoverlapping blocks, and CS is applied to each block independently; thus, a larger number of blocks results in better image quality. However, using a larger number of blocks consumes more memory and increases the computational complexity. Suppose that $x_{i}$ is a vector representing the i-th block of the input image and that the corresponding sampling vector $y_{i}$ is defined as follows [20]:

(11) $$y_{i}=\varphi_{i}x_{i},$$

where the dimensions of $\varphi_{i}$ are $AS_{i}\times \;N_{B}{}^{2}$, $AS_{i}$ is the corresponding number of samples of the i-th block, and N is the size of the entire image in vector form. Thus, the measurement matrix φ of the entire image x is defined as follows:

(12) $$\varphi =\left[\begin{array}{c} \begin{array}{cccc} \varphi_{1} & 0 & \cdots & 0 \end{array}\\ \begin{array}{cccc} 0 & \varphi_{2} & \cdots & 0 \end{array}\\ \begin{array}{cccc} \vdots \;\;\;\; & \vdots \;\; & \ddots \; & \vdots \end{array}\\ \begin{array}{cccc} 0 & \cdots & 0 & \varphi_{TB} \end{array}\; \end{array}\right].$$

Note that the reconstruction of each block requires only $\varphi_{i}$ and that all blocks can then be combined to reconstruct the original image. Consequently, BCS requires less memory and computational power than implementing CS for the complete image.

After the adaptive sampling has been derived for each block and CS measurements have been conducted to adaptively reduce the sampling for each block, the result is a set of extremely small compressed blocks. The compressed blocks are then transmitted from one SN to another in a hop-by-hop fashion until they finally arrive at the BS.

### 3.6. Image Reconstruction

As soon as each block arrives at the BS, that block is reconstructed using an image reconstruction algorithm. After all the blocks have been reconstructed, the reconstructed image is restored from all the reconstructed blocks.

In CS, image reconstruction is the process of recovering each image block from a very limited K-sparse sampling resulting from the CS measurement implementation. Many image signal reconstruction algorithms exist, as discussed above. However, BCS-SPL has attracted many researchers in the signal processing field due to its effectiveness and efficiency in improving the reconstructed image quality by eliminating blocking artifacts, and it is also suitable for real-time applications [38]. Therefore, in our proposed architecture, BCS-SPL is improved by using an FLS to enhance the quality of the reconstructed image. This subsection explains both the original BCS-SPL algorithm and our proposed reconstruction algorithm in detail.

#### 3.6.1. BCS with Smoothed Projected Landweber Reconstruction (BCS-SPL)

Here, we discuss a traditional BCS-SPL as a baseline and then discuss its enhancement with a fuzzy-based approach (see the next subsection). On the decoder side, in the image reconstruction stage, the original signal is recovered from the samples through iterative projection and hard thresholding. An initial approximation is calculated as follows:

(13) $$x^{0}=\varphi^{T}y$$

The projected Landweber approach, corresponding to the update process described in Equation (14), attempts to correct the signal $x^{r+1}\;$ from the measurement *y*:

(14) $${\hat{\hat{x}}}^{\left(r\right)}={\hat{x}}^{\left(r\right)}+\;\varphi^{T}\left(y-\varphi {\hat{x}}^{\left(r\right)}\right),$$

where ${\hat{\hat{x}}}^{\left(r\right)}$ and ${\hat{\hat{x}}}^{\left(r+1\right)}$ denote the data before and after the projected Landweber estimation, respectively.

The projected Landweber process is applied twice before the termination condition is checked. The first application occurs immediately after the blocking artifacts have been reduced in the provisionally reconstructed image using the Wiener filter, and the second occurs after artifacts have been reduced via hard thresholding in the DCT domain. The pseudocode for the BCS-SPL algorithm is provided in Algorithm 2.
**Algorithm 2** BCS-SPL Reconstruction Algorithm [38]**Input:** The measurement matrix for the i-th block: $\varphi_{i}$, a constant used to control the convergence speed: λ, the measurement: y, signal: $x^{\left(r\right)}$, 2D transform: ψ **Output:** signal: $x^{\left(r+1\right)}$ 1: ${\hat{x}}^{\left(r\right)}=Wiener\left(x^{\left(r\right)}\right)$ 2: **for each** block i 3: ${\hat{\hat{x}}}_{i}^{\left(r\right)}={\hat{x}}_{i}^{\left(r\right)}+\;\varphi_{i}^{T}\left(y_{i}-\varphi_{i}{\hat{x}}_{i}^{\left(r\right)}\right)$ 4: **end for** 5: ${\overset{ˇ}{\overset{ˇ}{x}}}^{\left(r\right)}=\psi {\hat{\hat{x}}}^{\left(r\right)}$ 6: ${\overset{ˇ}{x}}^{\left(r\right)}=Threshold\left({\overset{ˇ}{\overset{ˇ}{x}}}^{\left(r\right)},\lambda \right)$ 7: ${\bar{x}}^{\left(r\right)}=\psi^{-1}{\overset{ˇ}{x}}^{\left(r\right)}$ 8: **for each** block i 9: $x_{i}^{\left(r+1\right)}={\bar{x}}_{i}^{\left(r\right)}+\;\varphi_{i}^{T}\left(y_{i}-\varphi_{i}{\bar{x}}_{i}^{\left(r\right)}\right)$ 10: **end for**

In Algorithm 2, $\varphi_{i}$ is the measurement matrix for the i-th block, Wiener(.) represents the pixelwise adaptive Wiener filter with a neighborhood of 3×3, and Threshold(.) is the thresholding process shown below:

(15) $${\overset{ˇ}{x}}^{\left(r+1\right)}=\{\begin{array}{c} {\overset{ˇ}{\overset{ˇ}{x}}}^{\left(r\right)},\;{\overset{ˇ}{\overset{ˇ}{x}}}^{\left(r+1\right)}>\tau^{\left(r\right)}\\ 0,\;else \end{array}$$

where $\tau^{\left(r\right)}$ is the threshold value in iteration r. Its formula is

(16) $$\tau^{\left(r\right)}=\lambda \frac{median\left\{\left|{\overset{ˇ}{\overset{ˇ}{x}}}^{\left(r\right)}\right|\right\}}{0.6745}\sqrt{2ln\;L}$$

where λ is a constant used to control the convergence speed and L is the number of transform coefficients.

Although BCS-SPL achieves a good reconstructed image quality, the original method itself still has some problems since the speed of convergence, λ, is fixed and is thus not suitable for all sampling rates or all types of images. Therefore, we propose a fuzzy BCS-SPL reconstruction algorithm in which the speed of convergence is adaptively adjusted using an FLS.

#### 3.6.2. Fuzzy BCS-SPL Reconstruction

The results of extensive experiments show that the quality of BCS-SPL reconstruction can be improved by adjusting λ, which is a constant that controls the convergence speed. During our performance evaluation, we also found that the optimal value of λ depends on the feature fraction in Equation (6) and the sampling rate. As a result, we developed an FLS to determine the best λ value for a specific image and sampling rate. Our FLS has 2 fuzzy input variables, the first of which is the feature fraction, which can take 7 linguistic values (Extremely Low (XL), Very Low, Low, Medium, High, Very High, and Extremely High (XH)). A trapezoidal membership function is applied to all these values.

**Figure 8:**
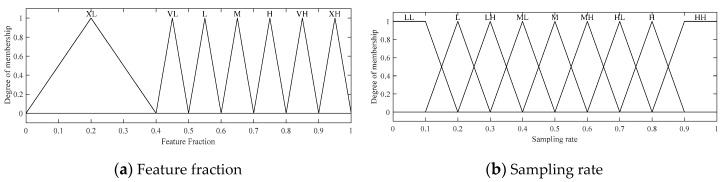
Two fuzzy input variables for the fuzzy BCS-SPL algorithm: (LL = Low Low, L = Low, LH = Low High, ML = Medium Low, M = Medium, MH = Medium High, HL = High Low, H = High, HH = High High, XL = Extremely Low, VL = Very Low, L = Low, M = Medium, H = High, VH = Very High, XH = Extremely High).

The second input variable in this FLS is the sampling rate is, which has 9 linguistic values (Low Low, Low, Low High, Medium Low, Medium, Medium High, High Low, High, and High High), as shown in Figure 8a. A triangular membership function is used for Low Low and High High, while a trapezoidal membership function is applied to the other values, as shown in Figure 8b.

**Figure 9:**
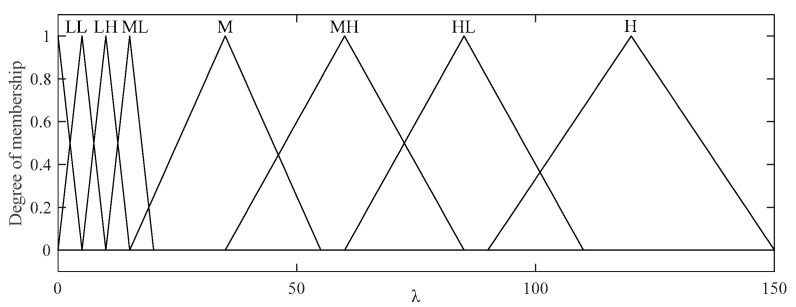
The fuzzy output for λ. (LL = Low Low, L = Low, LH = Low High, ML = Medium Low, M = Medium, MH = Medium High, HL = High Low, H = High).

The fuzzy inference process relies on 63 fuzzy rules generated from these two fuzzy input variables and one output variable, as described in Table 4. Analogous to the abovementioned fuzzy rules, these rules are adopted from the possible rules that were implemented. The output, as depicted in Figure 9, is λ.

In our proposed architecture, the convergence speed is controlled by the feature selection threshold (FST) as discussed in Section 3.4.2. Increasing or decreasing the value of FST affects both the convergence speed and the quality of the reconstructed image. Determining the best FST requires multiple experiments on many images, as will be discussed in the experimental section.

**Remark 1.**  *The triangular and trapezoidal membership functions perform better than do other membership functions, as shown by tests performed using various membership functions to investigate the resulting stability of the fuzzy system [54]. These membership functions are effective and frequently used for two reasons. First, they are computationally highly efficient since their membership calculations are linear. Therefore, they can be used to process large volumes of data, such as images. Second, the triangular and trapezoidal functions are easily visually understood and offer an environment that is reasonably conducive to human-in-the-loop knowledge acquisition [51]. Consequently, we adopt triangular and trapezoidal membership functions in this study.*

**Remark 2.**  *The proposed reconstruction method is improved relative to the work presented in [38] and extended to a more flexible solution by reducing the computational complexity and improving the reconstructed image quality. The performance and reliability of our algorithm are enhanced through the use of an FLS to adaptively determine the convergence speed of the BCS-SPL reconstruction algorithm based on the image characteristics and the sampling rate. The convergence speed determines the number of iterations of the BCS-SPL algorithm required to reconstruct the image. A large number of iterations increases the computational complexity without guaranteeing a better reconstructed image quality, while a small number of iterations reduces the complexity but can result in an unsatisfactory image reconstruction. The conventional BCS-SPL algorithm uses a fixed convergence speed for all blocks in the image. Utilizing an FLS based on the image features and sampling rate to determine a suitable convergence speed for each block individually helps address these problems. A small number of iterations can be used for less important blocks, while a larger number of iterations can be used for more important blocks. The complexity of our reconstruction algorithm is discussed in the next section.*

## 4. Experimental Results and Discussion

### 4.1. Experimental Settings

**Figure 10:**
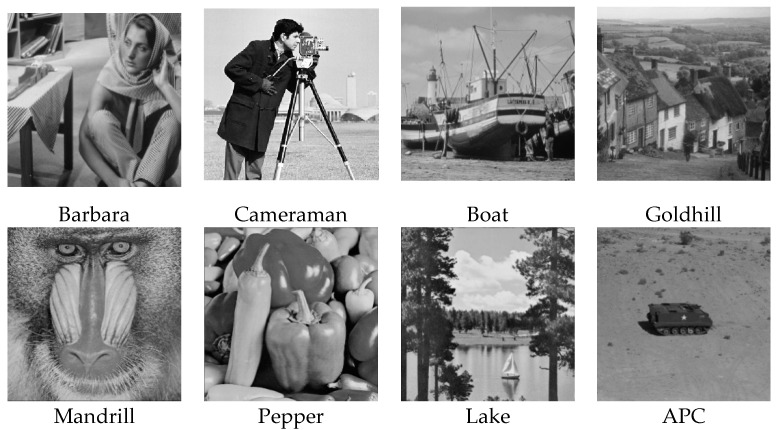
Standard test images [44].

The algorithms were simulated using MATLAB 2017 on a computer equipped with a 64-bit Windows 10 Pro operating system and an Intel (R) Core (TM) i5–7400 CPU @ 3.0 GHz and 16 GB of RAM. While other ABCS studies [13,16,23,38,55] used only five or six grayscale standard images in their experiments, we used eight grayscale standard images [44] depicting different categories (i.e., human, animal, fruit, landscape, building, and vehicle), as shown in Figure 10, in our simulations to demonstrate the effectiveness of our proposed architecture. We consider grayscale images instead of color images because two-thirds of the measurement can be further compressed and the complexity can be reduced dramatically; thus, the lifetime of WMSNs can be prolonged [56].

In our experiments, the sampling rate was varied from 0.1 to 0.9 with a step size of 0.1. Based on many experiments on different images, we found that our proposed architecture produces optimal results when the feature selection threshold (FST) is set to 0.1. We compared our proposed fuzzy ABCS architecture (FABCS) with the traditional BCS algorithm [20] and three state-of-the-art algorithms, namely, SABCS [31], STD-ABCS [14], and saliency-based adaptive BCS for images using measurement contrast (SABCS-MC) [16]. Similar to previous studies, because CS is performed using a random matrix, we conducted five trials and averaged the results [14,16,31]. The simulation parameters are summarized in Table 5.

There are two types of evaluation metrics, i.e., objective and subjective. For comparison purposes in our objective evaluation, we adopted the well-known peak-signal-to-noise ratio (PSNR) image quality metric to measure the performance of our proposed architecture. A higher PSNR value indicates a better reconstructed image quality. The PSNR is defined as follows [57]:

(17) $$PSNR=10log10\frac{MG^{2}}{MSE}$$

where MG is the maximum grayscale value of the image and MSE is the mean squared error, which represents the mean of the cumulative squared error between the reconstructed and original images.

A feature selection threshold (FST), as discussed in Section 3.4.2, is used to select the feature type to be used for sampling allocation for each block in our proposed architecture. Therefore, it is important to find the optimal FST value that can yield the best reconstructed image quality. To this end, we conducted many experiments on different images at various sampling rates from 0.1 to 0.9 and FST values from 0 to 1. Based on the results of these experiments, we can conclude that our proposed method produces the optimal results when the threshold is 0.1 for all test images and sampling rates. Next, we present the image reconstruction results of our proposed method.

### 4.2. Experimental Results

In this section, we evaluate the quality of the reconstructed images. An objective evaluation yields specific results based on mathematical formulas, while a subjective evaluation is generally based on observer perceptions when evaluating the quality of a reconstructed image [58].

**Figure 11:**
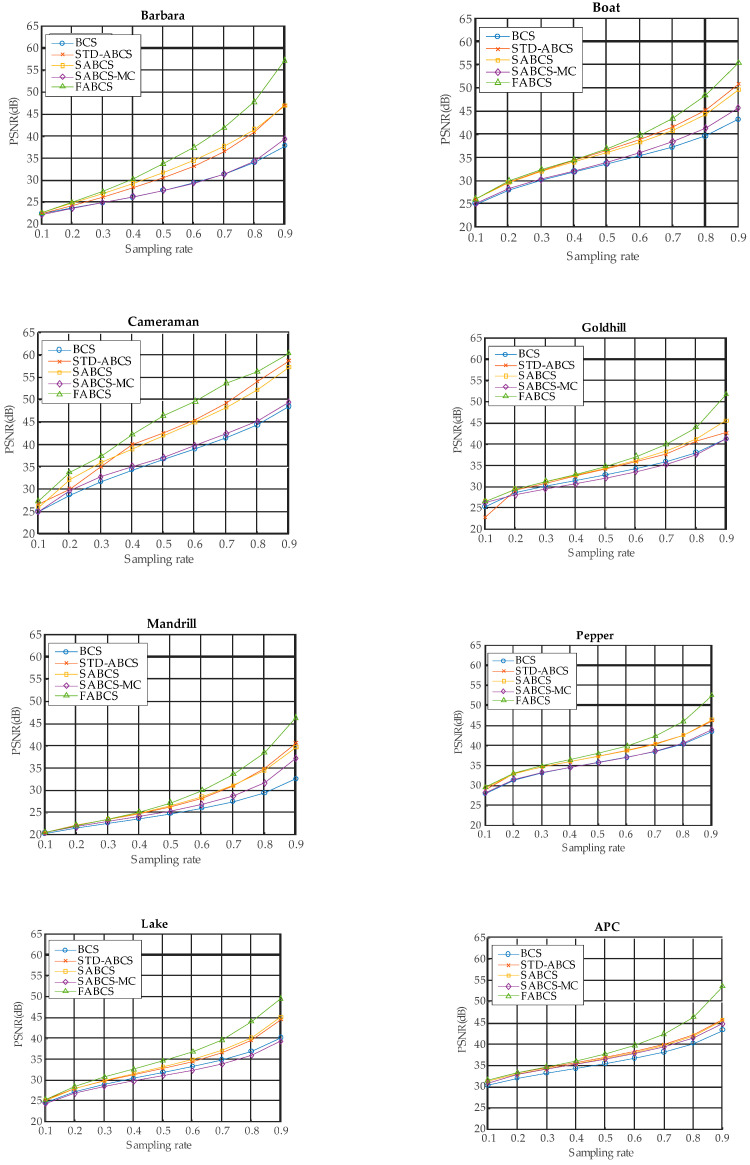
The experimental results for a block size of 16 × 16.

**Figure 12:**
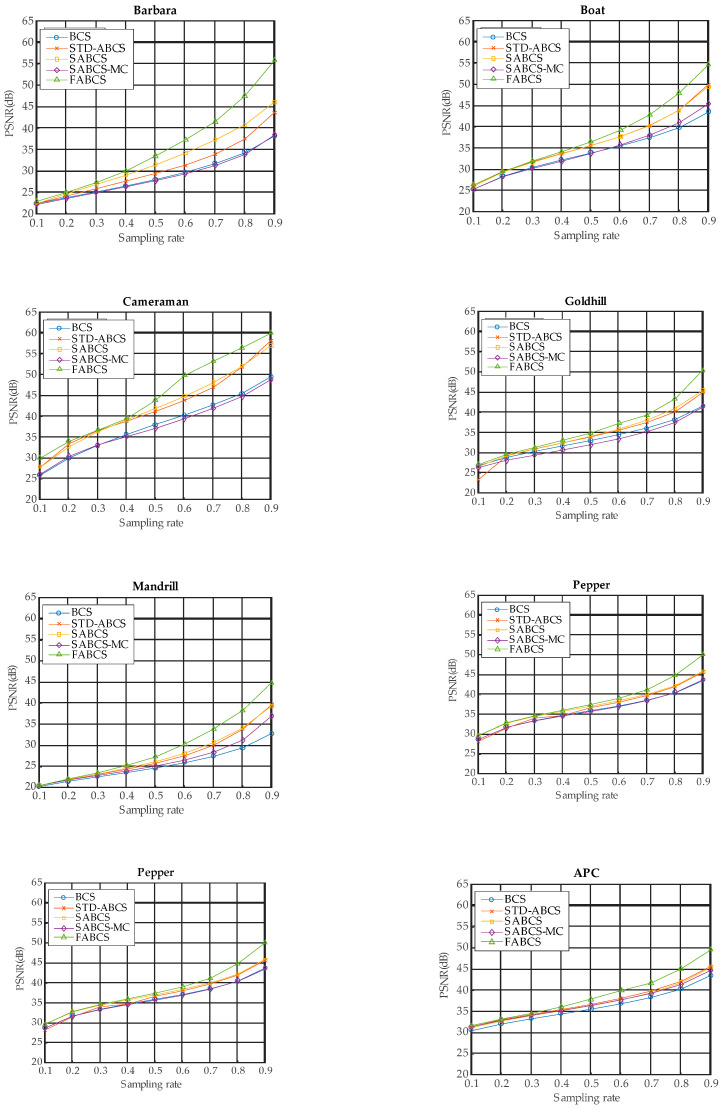
The experimental results for a block size of 32 × 32.

The experimental results for block sizes of 16 × 16 and 32 × 32 are presented in Figure 11 and Figure 12, respectively. For the 16 × 16 block size, on all test images, the BCS algorithm does not yield better results than our proposed architecture or some existing ABCS algorithms. However, BCS performs better than does SABCS-MC on the Cameraman, Goldhill, and Lake test images. For the 32 × 32 block size, the BCS algorithm produces the lowest image reconstruction quality compared with all of the other ABCS algorithms and our proposed architecture at all sampling rates on the Mandrill and APC test images. However, BCS performs better than the STD-BCS algorithm on the Goldhill image at a sampling rate of 0.1 and on the Peppers image at sampling rates of 0.1 and 0.2. Moreover, the BCS algorithm achieves a higher PSNR than does SABCS-MC on the Barbara, Boat, Cameraman, Goldhill, Peppers, and Lake images at most sampling rates.

For both block sizes, the STD-ABCS algorithm yields improved reconstructed images on the Boat, Cameraman, and APC images at the majority of sampling rates compared with BCS and the other ABCS algorithms. These results show that STD-ABCS results in better image reconstruction for low-intensity images.

On the other hand, for both block sizes, SABCS achieves better results than do BCS and the other ABCS algorithms on high-intensity images such as Barbara and Peppers at all sampling rates and on the Goldhill and Mandrill images at most sampling rates.

The results of the SABCS-MC algorithm for all block sizes and test images are lower than those of the BCS and ABCS algorithms as well as our proposed architecture.

For both block sizes, our proposed architecture yields satisfactory results at very low sampling rates of 0.1 and 0.2 and achieves the best performance at medium and high sampling rates between 0.3 and 0.9 on the following test images: Barbara, Cameraman, Mandrill, and Lake. For Boat, Goldhill, Peppers and APC, our proposed architecture produces acceptable results at all sampling rates.

These experimental results show the outstanding performance of our proposed FABCS architecture and clearly indicate that our proposed architecture offers better image reconstruction quality than both the conventional BCS algorithm and state-of-the-art ABCS algorithms. The results show that as the sampling rate increases, our proposed architecture achieves better PSNR results. This is because at a high sampling rate, the number of samples is large, and our proposed architecture can properly allocate the sampling to each block. Overall, for the various test images and sampling rates, our proposed FABCS architecture yields average PSNR values that are 5.21 dB, 2.56 dB, 2.55 dB, and 4.22 dB better than those of BCS, STD-ABCS, SABCS, and SABCS-MC, respectively. Moreover, the experimental results show that our proposed architecture produces better results when the block size is small.

In addition, we subjectively evaluated the ABCS algorithms based on visualizations of the reconstructed images. Table 6 shows sample reconstructed images (cameraman [44]) for all the ABCS algorithms (32 × 32 block size) used in this study at various sampling rates (e.g., 0.1, 0.3 and 0.5). At low sampling rates, a limited number of samplings exist for ABCS; therefore, most of the information is lost. Consequently, the distortion rate of the reconstructed images is high. As the sampling rate increases, the quality of the reconstructed images improves.

Based on the results in Table 6, because the BCS algorithm assigns the same fixed sampling to all blocks without considering whether the blocks contain vital information, the rate of distortion is very high at the sampling rate of 0.1. The quality of the reconstructed images is improves slightly at sampling rates of 0.3 and 0.5; however, their quality is still rather low compared with those produced by the ABCS algorithms and our proposed architecture because the reconstructed images lose many structural details.

In the STD-ABCS algorithm, adaptive sampling is assigned based on the standard deviation. The results of the STD-ABCS algorithm are substantially improved compared with BCS and SABCS-MC and slightly enhanced compared with SABCS. However, at a sampling rate of 0.1, blocking artifacts clearly appear at the top of the reconstructed image. The images reconstructed using SABCS are superior to those reconstructed using BCS and SABCS-MC; unfortunately, the structural details of the images reconstructed by SABCS are slightly worse than those obtained using STD-ABCS and our proposed architecture.

The SABCS-MC algorithm results in a reconstructed image only outperforms BCS because many blocking artifacts are visible around the edges of the reconstructed image, and some of the structural details of the reconstructed image are lost.

As shown in Table 6, compared with the BCS and the other ABCS algorithms, our proposed FABCS architecture more effectively reduces blocking artifacts and retains the structural details of the reconstructed images, such as texture, corners and edges; therefore, our proposed architecture ensures superior subjective visual quality.

## 5. Conclusions

CS is an effective, simple, and low-complexity signal coding algorithm suitable for implementation in resource-constrained WMSNs to enhance image transmission. In this article, we presented the fuzzy adaptive block compressed sensing architecture (FABCS), which improves reconstructed image quality while maintaining low complexity and memory usage that are suitable for execution by SNs. Our algorithm uses an FLS to select the most suitable feature type to use as the basis for adaptive sampling allocation for each block. The base sampling is calculated and used as the base sampling configuration for each block before additional adaptive sampling is allocated based on the selected feature type. When the total number of samples allocated to a block exceeds its size, our proposed oversampling adjustment algorithm eliminates oversampled blocks.

For image reconstruction, we propose the fuzzy BCS-SPL algorithm, which adjusts the convergence speed of BCS-SPL using an FLS. The experimental results show that the proposed FABCS architecture yields better-quality reconstructed images than do the existing methods, including BCS, SABCS, STD-ABCS, and SABCS-MC, particularly as the sampling rate increases. Moreover, FABCS works even better for low-intensity images. However, several interesting questions remain to be solved in our future work, as described below.

First, we plan to leverage the results of the current study and design a new architecture suitable for color image applications because color images include more information than do grayscale images. Second, we plan to investigate the possibility of using an irregular or adaptive block size, which may produce better results than a fixed block size. Further work may also involve investigating other distributions, e.g., asymmetric and convergent distributions [59,60]. We also plan to investigate neutrosophic logic [61] in future studies. Note that although CS derivatives are suitable for on use on limited-resource devices, a deep analysis of space/time usage should also be conducted when more advanced techniques have been embedded. Moreover, considering that multimedia signals include not only images but also video, we plan to develop an adaptive CS algorithm that can be applied to such data. Finally, we are also interested in finding other, more effective multimedia content features for use in future adaptive-sampling BCS algorithms.

## Figures and Tables

**Table 1:** The overall notations used in this study.

Notation	Description
C	2D discrete cosine transform (DCT)
$C^{-1}$	2D DCT inverse transform
sign(.)	Signum function
SM	Saliency map
G	Gaussian low-pass filter
STD	Standard deviation
*Sal*	Saliency
$x_{i}$	The *i*-th image block
$x_{i}\left(j\right)$	The value of the *j*-th pixel of the *i*-th image block
$N_{B}$	The size of the vector representing the block
mean(.)	The function used to calculate the mean value.
$FSB_{i}$	The feature selection computation for the *i*-th block
FSC	The fuzzy output of the FLS for feature selection
FST	The constant feature selection threshold
$NFSB_{Sal}$	The number of blocks for which saliency was selected as the preferred feature type
TB	The total number of blocks in the image.
FF	The feature fraction
$INT_{i}$	The intensity of the *i*-th block
MG	The maximum grayscale value in the image (e.g., 255)
$\;M_{i}^{0}$	The base sampling for the *i*-th block
$AS_{i}$	The adaptive sampling for the *i*-th block
$F_{i}$	The feature (e.g., standard deviation or saliency) for the *i*-th block
N	The size of the image vector
rnd(.)	The function that rounds its argument to the nearest integer
$y_{i}$	The sampling vector of the *i*-th block
$\varphi_{i}$	The measurement matrix of *i*-th block

**Table 2:** Fuzzy rules regarding the fuzzy cost for feature selection.

Standard Deviation	Saliency	*FSFuzzyCost*
Very Low	Very Low	Low Low
Very Low	Low	Low
Very Low	Medium	Low High
Very Low	High	Medium Low
Very Low	Very High	Medium
Low	Very Low	Low
Low	Low	Low High
Low	Medium	Medium Low
Low	High	Medium
Low	Very High	Medium High
Medium	Very Low	Low High
Medium	Low	Medium Low
Medium	Medium	Medium
Medium	High	Medium High
Medium	Very High	High Low
High	Very Low	Medium Low
High	Low	Medium
High	Medium	Medium High
High	High	High Low
High	Very High	High
Very High	Very Low	Medium
Very High	Low	Medium High
Very High	Medium	High Low
Very High	High	High
Very High	Very High	High High

**Table 3:** Fuzzy rules.

Intensity	Sampling Rate	Weight
Low	Very Low	Low
Low	Low	Very Low
Low	Medium	Very Low
Low	High	Very Low
Low	Very High	Very Low
High	Very Low	Very High
High	Low	High
High	Medium	Medium
High	Very High	Low
High	Very High	Very Low

**Table 4:** Fuzzy rules for the fuzzy cost for λ.

Feature	Sampling Rate	λ
Extremely Low	Low Low	Medium
Extremely Low	Low	Medium Low
Extremely Low	Low High	Medium Low
Extremely Low	Medium Low	Low High
Extremely Low	Medium	Low High
Extremely Low	Medium High	Low
Extremely Low	High Low	Low
Extremely Low	High	Low Low
Extremely Low	High High	Low Low
Very Low	Low Low	High
Very Low	Low	High Low
Very Low	Low High	High Low
Very Low	Medium Low	Medium High
Very Low	Medium	Medium High
Very Low	Medium High	Medium
Very Low	High Low	Medium
Very Low	High	Low Low
Very Low	High High	Low Low
Low	Low Low	Medium
Low	Low	Medium Low
Low	Low High	Low High
Low	Medium Low	Low
Low	Medium	Low
Low	Medium High	Low
Low	High Low	Low Low
Low	High	Low Low
Low	High High	Low Low
Medium	Low Low	Medium
Medium	Low	Medium Low
Medium	Low High	Low High
Medium	Medium Low	Low
Medium	Medium	Low
Medium	Medium High	Low
Medium	High Low	Low Low
Medium	High	Low Low
Medium	High High	Low Low
High	Low Low	Medium
High	Low	Medium Low
High	Low High	Low High
High	Medium Low	Low
High	Medium	Low
High	Medium High	Low
High	High Low	Low
High	High	Low Low
High	High High	Low Low
Very High	Low Low	Medium Low
Very High	Low	Medium Low
Very High	Low High	Low
Very High	Medium Low	Low
Very High	Medium	Low Low
Very High	Medium High	Low Low
Very High	High Low	Low Low
Very High	High	Low Low
Very High	High High	Low Low
Extremely High	Low Low	Medium High
Extremely High	Low	Medium
Extremely High	Low High	Medium Low
Extremely High	Medium Low	Low High
Extremely High	Medium	Low High
Extremely High	Medium High	Low
Extremely High	High Low	Low
Extremely High	High	Low Low
Extremely High	High High	Low Low

**Table 5:** Simulation parameters.

Parameter	Symbol	Value
Block size	$N_{B}$	16 × 16, 32 × 32 [14,16]
Total number of blocks	TB	256 [14,16]
Image size	N	512 × 512 pixels (grayscale) [14,16]
Feature selection threshold	FST	0.1
BCS-SPL reconstruction algorithm		
Maximum iterations		200
Tolerance value	TOL	0.0001
DWT level	L	5

**Table 6:** Image of the ABCS algorithm at different sampling rates (32 x 32 block size).

Method	Sampling Rate		
	0.1	0.3	0.5
BCS	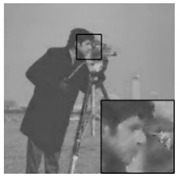	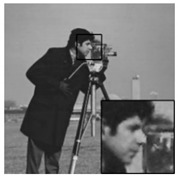	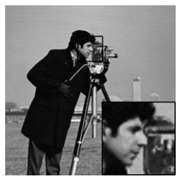
STD-BCS	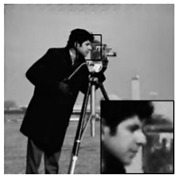	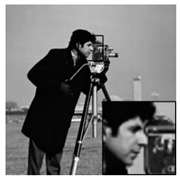	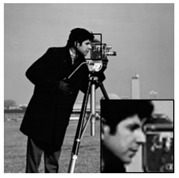
SABCS	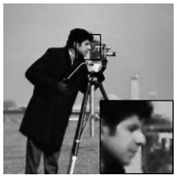	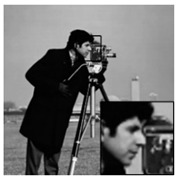	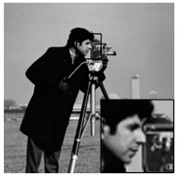
SACS-MC	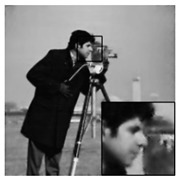	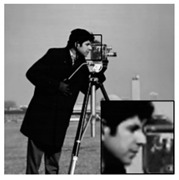	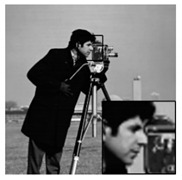
FABCS